# Methyl *trans*-(±)-1-oxo-2-phenethyl-3-(thio­phen-2-yl)-1,2,3,4-tetra­hydro­isoquinoline-4-carboxyl­ate

**DOI:** 10.1107/S1600536809017383

**Published:** 2009-05-14

**Authors:** Mehmet Akkurt, Selvi Karaca, Milen G. Bogdanov, Meglena I. Kandinska, Orhan Büyükgüngör

**Affiliations:** a Department of Physics, Faculty of Arts and Sciences, Erciyes University, 38039 Kayseri, Turkey; b Faculty of Chemistry, University of Sofia, 1 James Bourchier Boulevard, 1164 Sofia, Bulgaria; c Department of Physics, Faculty of Arts and Sciences, Ondokuz Mayıs University, 55139 Samsun, Turkey

## Abstract

In the title compound, C_23_H_21_NO_3_S, the piperidine ring of the tetra­hydro­isoquinolinone unit adopts a screw-boat conformation. The thio­phene ring is disordered in a 0.700 (3):0.300 (3) ratio by an approximate 180° rotation of the ring around the C—C bond linking the ring to the tetra­hydro­isoquinolinone unit. The benzene ring of the tetra­hydro­isoquinolinone unit makes dihedral angles of 83.1 (2) and 62.38 (11)° with the major occupancy thio­phene ring and the phenyl ring, respectively. The dihedral angle between the phenyl ring and the thio­phene ring is 71.0 (2)°. In the crystal structure, mol­ecules are linked together by inter­molecular C—H⋯O and C—H⋯π inter­actions.

## Related literature

For background to the biological and pharmacological applications of compounds containing a tetra­hydro­isoquinoline fragment, see: Bogdanov *et al.* (2007 ▶); Burdzhiev & Stanoeva (2006 ▶); Gitto *et al.* (2008 ▶); Humphries *et al.* (2009 ▶); Kandinska *et al.* (2006 ▶); Rothweiler *et al.* (2008 ▶). For reference structural data, see: Allen *et al.* (1987 ▶); Akkurt *et al.* (2008 ▶). For ring conformations, see: Cremer & Pople (1975 ▶).
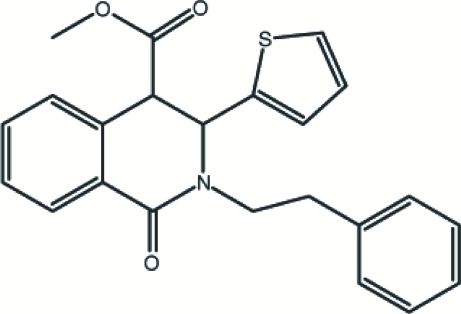


## Experimental

### 

#### Crystal data

C_23_H_21_NO_3_S
*M* *_r_* = 391.48
Monoclinic, 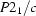

*a* = 8.8841 (3) Å
*b* = 30.7095 (13) Å
*c* = 7.5757 (3) Å
β = 105.472 (3)°
*V* = 1991.95 (14) Å^3^
*Z* = 4
Mo *K*α radiation
μ = 0.19 mm^−1^
*T* = 296 K
0.65 × 0.47 × 0.22 mm

#### Data collection

Stoe IPDS 2 diffractometer
Absorption correction: integration (*X-RED32*; Stoe & Cie, 2002 ▶) *T*_min_ = 0.889, *T*_max_ = 0.960
13399 measured reflections
3986 independent reflections
3154 reflections with *I* > 2σ(*I*)
*R*_int_ = 0.036

#### Refinement

*R*[*F* ^2^ > 2σ(*F* ^2^)] = 0.048
*wR*(*F* ^2^) = 0.137
*S* = 1.04
3986 reflections
273 parameters
36 restraints
H-atom parameters constrained
Δρ_max_ = 0.32 e Å^−3^
Δρ_min_ = −0.23 e Å^−3^

### 

Data collection: *X-AREA* (Stoe & Cie, 2002 ▶); cell refinement: *X-AREA*; data reduction: *X-RED32* (Stoe & Cie, 2002 ▶); program(s) used to solve structure: *SIR97* (Altomare *et al.*, 1999 ▶); program(s) used to refine structure: *SHELXL97* (Sheldrick, 2008 ▶); molecular graphics: *ORTEP-3 for Windows* (Farrugia, 1997 ▶); software used to prepare material for publication: *WinGX* (Farrugia, 1999 ▶).

## Supplementary Material

Crystal structure: contains datablocks global, I. DOI: 10.1107/S1600536809017383/is2413sup1.cif

Structure factors: contains datablocks I. DOI: 10.1107/S1600536809017383/is2413Isup2.hkl

Additional supplementary materials:  crystallographic information; 3D view; checkCIF report

## Figures and Tables

**Table 1:** Hydrogen-bond geometry (Å, °)

*D*—H⋯*A*	*D*—H	H⋯*A*	*D*⋯*A*	*D*—H⋯*A*
C2—H2⋯O1^i^	0.93	2.57	3.401 (3)	149
C15—H15⋯O1^ii^	0.93	2.55	3.420 (3)	155
C3—H3⋯*Cg*1^iii^	0.93	2.78	3.688 (3)	165
C3—H3⋯*Cg*2^iii^	0.93	2.77	3.688 (4)	167
C19—H19⋯*Cg*3^iv^	0.93	2.89	3.692 (3)	145

Symmetry codes: (i) 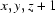; (ii) 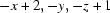; (iii) 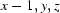; (iv) 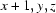. *Cg*1, *Cg*2 and *Cg*3 are centroids of the S1*A*/C12/C13*A*/C14/C15, S1*B*/C12/C13*B*/C14/C15 and C1–C6 rings, respectively.

**Fig. 1.**
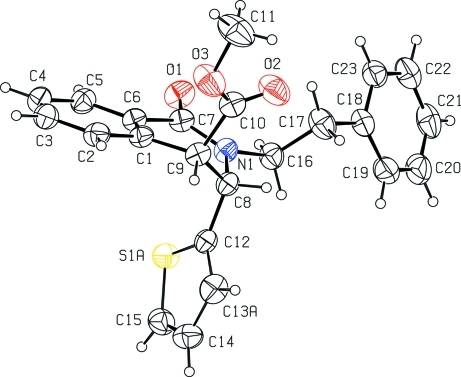
View of the title molecule, (I), with the atom-labelling scheme. Displacement ellipsoids are drawn at the 30% probability level. H atoms are shown as spheres of arbitrary radius. The minor component of the disorder groups has been omitted.

**Fig. 2.**
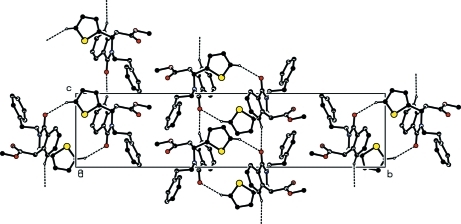
Part of the crystal structure of (I), viewed along the a axis. Dashed lines show intermolecular C—H···O interactions. H atoms not involved in hydrogen bonding and the minor component of the disorder groups have been omitted for clarity.

## supplementary crystallographic information

### Comment

Compounds containing tetrahydroisoquinoline fragment in their structure display a broad spectrum of biological activities. In particular, derivatives of this type have been recently recognized as being G-protein-coupled receptor 40 (GPR40) antagonists (Humphries *et al.*2009), inhibitors of the MDM2–p53 interaction (Rothweiler *et al.*, 2008), potent anticonvulsant agents (Gitto *et al.*, 2008), *etc*. Thus, the title compound (I) was synthesized as a part of our ongoing program related to anhydride-based synthesis of new heterocyclic compounds with potential pharmacological activities (Bogdanov *et al.*, 2007; Burdzhiev & Stanoeva, 2006; Kandinska *et al.*, 2006).

In the title molecule, (I), the thiophene ring is disordered over two sites and the major component of the disorder labelled with suffix A is shown in Fig. 1. The disorder corresponds to a rotation of approximately 180° rotation about the single C—C bond to which it is attached. All the bond lengths and angles of (I) are in normal ranges (Allen *et al.*, 1987; Akkurt *et al.*, 2008). The six-membered piperidine ring (N1/C1/C6–C9) of 3,4-dihydroisoquinolinone ring system adopts a screw-boat conformation, as shown with the Cremer–Pople puckering parameters [Cremer & Pople, 1975; Q_T_ = 0.4620 (18) Å, θ = 114.5 (2)° and φ = 90.8 (23)°]. The benzene ring (C1–C6) of 3,4-dihydroisoquinolinone ring system is essentially planar, with an r.m.s. deviation of 0.002 (2) Å for C2 and C5. This benzene ring makes dihedral angles of 83.1 (2), 83.1 (4) and 62.38 (11)°, with the thiophene rings A (C12/C13A/C14/C15/S1A) and B (C12/C13B/C14/C15/S1B), and the phenyl ring C (C18–C23), respectively. The dihedral angles between the phenyl ring C and the thiophene rings A and B are C/A = 71.0 (2) and C/B = 70.4 (4)°, respectively.

In the crystal structure, molecules are linked together by intermolecular C—H···O interactions (Table 1 and Fig. 2). The crystal structure is further stabilized by intermolecular C—H···π interactions (Table 1).

### Experimental

The title compound (I) was synthesized by esterification reaction of *trans*-1-oxo-2-phenethyl-3-(thiophen-2-yl)-1,2,3,4- tetrahydroisoquinoline-4-carboxylic acid (20 g, 0.053 mol) in the presence of H_2_SO_4_ (4.3 ml) in methanol. The reaction mixture was refluxed for 3 h. and then left over night. The colourless crystals were filtered and washed with water/methanol mixture yielding 18.6 g (90%) of (I). Single crystals were obtained by slow evaporation of a chloroform–ethyl acetate (3:1) solution of (I) at room temperature (mp 401–402 K). Analysis, calculated for C_23_H_21_NO_3_S (391.48): C 70.56, H 5.41 (%); found: C 70.24, H 5.38 (%). IR (CHCl_3_) 1650 cm^-1^ (C═O), 1740 cm^-1^ (C═O). The ^1^H NMR spectrum of (I) was obtained on a Bruker DRX-250 spectrometer at 250.13 MHz. Chemical shifts (δ) are expressed in parts per million (p.p.m.) from tetramethylsilane as an internal standard. ^1^H NMR (250 MHz, deuterochloroform) δ = 2.90–3.05 (2H, m, CH_2_-Phenyl), 3.18–3.30 (1H, m, CH_2_—N), 3.69 (1H, s, –OCH_3_), 4.05 (1H, s, H-4), 4.20–4.35 (1H, m, CH_2_—N), 5.60 (1H, s, H-3), 6.80 (2H, d, *J* = 5 Hz, H—Th), 7.10 (1H, d, *J* = 4 Hz, H—Th), 7.15–7.35 (7H, m, H—Ph), 7.45–7.55 (1H, m, H—Ph), 8.15–8.20 (1H, dd, H—Ph).

### Refinement

H atoms bound to C atoms were in geometrically generated positions and constrained to ride on their parent atoms [C—H = 0.93–0.98 Å and *U*_iso_(H) = 1.2 (1.5 for methyl groups) × *U*_eq_(C)]. The ratio of the refined occupancies for the major and minor components of the disordered thiophene ring is 0.700 (3):0.300 (3). Rigid-bond restrains were applied to the disordered atoms.

### Crystal data

**Table:**

C_23_H_21_NO_3_S	*F*(000) = 824
*M**_r_* = 391.48	*D*_x_ = 1.305 Mg m^−^^3^
Monoclinic, *P*2_1_/*c*	Mo *K*α radiation, λ = 0.71073 Å
Hall symbol: -P 2ybc	Cell parameters from 17858 reflections
*a* = 8.8841 (3) Å	θ = 1.3–26.8°
*b* = 30.7095 (13) Å	µ = 0.19 mm^−^^1^
*c* = 7.5757 (3) Å	*T* = 296 K
β = 105.472 (3)°	Block, colourless
*V* = 1991.95 (14) Å^3^	0.65 × 0.47 × 0.22 mm
*Z* = 4	

### Data collection

**Table:**

Stoe IPDS 2 diffractometer	3986 independent reflections
Radiation source: sealed X-ray tube, 12 x 0.4 mm long-fine focus	3154 reflections with *I* > 2σ(*I*)
plane graphite	*R*_int_ = 0.036
Detector resolution: 6.67 pixels mm^-1^	θ_max_ = 26.3°, θ_min_ = 1.3°
ω scans	*h* = −10→10
Absorption correction: integration (*X-RED32*; Stoe & Cie, 2002)	*k* = −38→38
*T*_min_ = 0.889, *T*_max_ = 0.960	*l* = −9→9
13399 measured reflections	

### Refinement

**Table:**

Refinement on *F*^2^	Primary atom site location: structure-invariant direct methods
Least-squares matrix: full	Secondary atom site location: difference Fourier map
*R*[*F*^2^ > 2σ(*F*^2^)] = 0.048	Hydrogen site location: inferred from neighbouring sites
*wR*(*F*^2^) = 0.137	H-atom parameters constrained
*S* = 1.04	*w* = 1/[σ^2^(*F*_o_^2^) + (0.0696*P*)^2^ + 0.315*P*] where *P* = (*F*_o_^2^ + 2*F*_c_^2^)/3
3986 reflections	(Δ/σ)_max_ < 0.001
273 parameters	Δρ_max_ = 0.32 e Å^−^^3^
36 restraints	Δρ_min_ = −0.22 e Å^−^^3^

### Special details

**Table:**

Geometry. Bond distances, angles *etc*. have been calculated using the rounded fractional coordinates. All su's are estimated from the variances of the (full) variance-covariance matrix. The cell e.s.d.'s are taken into account in the estimation of distances, angles and torsion angles
Refinement. Refinement on *F*^2^ for ALL reflections except those flagged by the user for potential systematic errors. Weighted *R*-factors *wR* and all goodnesses of fit *S* are based on *F*^2^, conventional *R*-factors *R* are based on *F*, with *F* set to zero for negative *F*^2^. The observed criterion of *F*^2^ > σ(*F*^2^) is used only for calculating -*R*-factor-obs *etc*. and is not relevant to the choice of reflections for refinement. *R*-factors based on *F*^2^ are statistically about twice as large as those based on *F*, and *R*-factors based on ALL data will be even larger.

### Fractional atomic coordinates and isotropic or equivalent isotropic displacement parameters (Å^2^)

**Table:**

	*x*	*y*	*z*	*U*_iso_*/*U*_eq_	Occ. (<1)
S1A	1.0536 (2)	0.02857 (4)	0.7180 (2)	0.0674 (4)	0.700 (3)
O1	0.78832 (17)	0.09928 (5)	0.29160 (17)	0.0760 (5)	
O2	0.8929 (2)	0.20317 (6)	0.7118 (3)	0.1030 (8)	
O3	0.7137 (2)	0.19259 (5)	0.8557 (3)	0.0992 (7)	
N1	0.96104 (17)	0.11653 (5)	0.5600 (2)	0.0562 (5)	
C1	0.7254 (2)	0.10320 (6)	0.7433 (2)	0.0546 (5)	
C2	0.6148 (2)	0.09167 (7)	0.8347 (3)	0.0645 (7)	
C3	0.4866 (2)	0.06686 (7)	0.7463 (3)	0.0725 (8)	
C4	0.4676 (2)	0.05369 (7)	0.5666 (3)	0.0729 (7)	
C5	0.5759 (2)	0.06508 (7)	0.4751 (3)	0.0656 (6)	
C6	0.7055 (2)	0.08982 (6)	0.5626 (2)	0.0547 (5)	
C7	0.8209 (2)	0.10222 (6)	0.4600 (2)	0.0568 (6)	
C8	1.0051 (2)	0.11775 (6)	0.7606 (2)	0.0547 (6)	
C9	0.8651 (2)	0.13115 (6)	0.8298 (2)	0.0563 (6)	
C10	0.8276 (2)	0.17975 (7)	0.7885 (3)	0.0637 (7)	
C11	0.6610 (4)	0.23722 (9)	0.8144 (5)	0.1154 (15)	
C12	1.0783 (2)	0.07522 (6)	0.8414 (2)	0.0532 (6)	
C13A	1.1728 (12)	0.0660 (3)	1.0108 (10)	0.075 (2)	0.700 (3)
C14	1.2240 (3)	0.02237 (8)	1.0421 (3)	0.0779 (8)	
C15	1.1645 (3)	0.00027 (7)	0.8887 (3)	0.0750 (8)	
C16	1.0833 (2)	0.12589 (7)	0.4683 (3)	0.0639 (7)	
C17	1.1146 (3)	0.17379 (7)	0.4507 (4)	0.0815 (9)	
C18	1.2171 (3)	0.18088 (6)	0.3240 (3)	0.0651 (7)	
C19	1.3667 (3)	0.16428 (7)	0.3613 (4)	0.0816 (8)	
C20	1.4523 (3)	0.16691 (8)	0.2327 (5)	0.0933 (10)	
C21	1.3917 (3)	0.18710 (8)	0.0716 (4)	0.0912 (11)	
C22	1.2473 (3)	0.20540 (7)	0.0333 (3)	0.0835 (9)	
C23	1.1594 (3)	0.20202 (7)	0.1584 (3)	0.0717 (7)	
S1B	1.1881 (8)	0.07244 (17)	1.0566 (8)	0.0679 (10)	0.300 (3)
C13B	1.076 (2)	0.0343 (5)	0.7659 (19)	0.102 (6)	0.300 (3)
H3	0.41300	0.05900	0.80780	0.0870*	
H4	0.38120	0.03710	0.50790	0.0870*	
H2	0.62690	0.10060	0.95500	0.0770*	
H8	1.08470	0.14040	0.79880	0.0660*	
H9	0.89210	0.12690	0.96280	0.0680*	
H5	0.56260	0.05620	0.35450	0.0790*	
H11B	0.57080	0.24250	0.85830	0.1730*	
H11C	0.63460	0.24170	0.68430	0.1730*	
H13A	1.20200	0.08730	1.10100	0.0890*	0.700 (3)
H14	1.28780	0.01120	1.15050	0.0930*	
H15	1.18410	−0.02920	0.87830	0.0900*	
H16A	1.05350	0.11310	0.34690	0.0770*	
H16B	1.17930	0.11190	0.53610	0.0770*	
H17A	1.01640	0.18900	0.40370	0.0980*	
H17B	1.16530	0.18560	0.57050	0.0980*	
H19	1.41080	0.15120	0.47400	0.0980*	
H20	1.55150	0.15470	0.25780	0.1120*	
H21	1.44920	0.18850	−0.01430	0.1100*	
H22	1.20770	0.22010	−0.07650	0.1000*	
H23	1.05990	0.21410	0.13070	0.0860*	
H11A	0.74300	0.25690	0.87320	0.1730*	
H13B	1.02290	0.02850	0.64490	0.1210*	0.300 (3)

### Atomic displacement parameters (Å^2^)

**Table:**

	*U*^11^	*U*^22^	*U*^33^	*U*^12^	*U*^13^	*U*^23^
S1A	0.0741 (9)	0.0596 (5)	0.0629 (8)	0.0032 (4)	0.0084 (6)	−0.0080 (4)
O1	0.0754 (9)	0.1048 (11)	0.0480 (7)	0.0044 (8)	0.0169 (6)	−0.0042 (7)
O2	0.1119 (14)	0.0757 (10)	0.1406 (16)	0.0198 (9)	0.0672 (13)	0.0246 (11)
O3	0.1201 (14)	0.0686 (9)	0.1341 (15)	0.0215 (9)	0.0780 (13)	0.0070 (9)
N1	0.0536 (8)	0.0661 (9)	0.0515 (8)	−0.0010 (7)	0.0186 (7)	−0.0004 (6)
C1	0.0504 (9)	0.0610 (10)	0.0527 (9)	0.0077 (8)	0.0143 (7)	0.0026 (8)
C2	0.0594 (11)	0.0758 (12)	0.0622 (11)	0.0094 (9)	0.0231 (9)	0.0077 (9)
C3	0.0590 (12)	0.0774 (13)	0.0877 (15)	0.0054 (10)	0.0309 (11)	0.0170 (11)
C4	0.0544 (11)	0.0699 (12)	0.0913 (15)	−0.0040 (9)	0.0142 (10)	0.0013 (11)
C5	0.0591 (11)	0.0673 (11)	0.0670 (11)	−0.0009 (9)	0.0111 (9)	−0.0068 (9)
C6	0.0516 (9)	0.0590 (9)	0.0529 (9)	0.0039 (7)	0.0131 (7)	−0.0001 (7)
C7	0.0572 (10)	0.0628 (10)	0.0505 (9)	0.0053 (8)	0.0146 (8)	−0.0006 (8)
C8	0.0514 (10)	0.0591 (10)	0.0532 (9)	−0.0018 (8)	0.0133 (8)	−0.0052 (7)
C9	0.0557 (10)	0.0636 (10)	0.0499 (9)	0.0058 (8)	0.0144 (8)	−0.0057 (8)
C10	0.0642 (12)	0.0695 (11)	0.0599 (11)	0.0055 (9)	0.0211 (9)	−0.0057 (9)
C11	0.153 (3)	0.0759 (16)	0.143 (3)	0.0394 (17)	0.084 (2)	0.0159 (16)
C12	0.0473 (9)	0.0595 (10)	0.0538 (10)	−0.0009 (7)	0.0155 (8)	−0.0058 (8)
C13A	0.082 (4)	0.076 (3)	0.066 (5)	−0.003 (2)	0.020 (3)	−0.023 (3)
C14	0.0747 (14)	0.0902 (15)	0.0677 (13)	0.0139 (12)	0.0170 (11)	0.0108 (11)
C15	0.0753 (13)	0.0624 (11)	0.0917 (15)	0.0072 (10)	0.0300 (12)	0.0033 (11)
C16	0.0637 (12)	0.0669 (11)	0.0694 (12)	−0.0003 (9)	0.0320 (10)	0.0008 (9)
C17	0.1019 (18)	0.0666 (12)	0.0921 (16)	0.0002 (11)	0.0537 (14)	0.0013 (11)
C18	0.0757 (13)	0.0521 (9)	0.0756 (12)	−0.0051 (9)	0.0342 (11)	0.0001 (9)
C19	0.0792 (15)	0.0727 (13)	0.0976 (16)	0.0014 (11)	0.0316 (13)	0.0182 (12)
C20	0.0753 (15)	0.0828 (15)	0.135 (2)	−0.0008 (12)	0.0510 (16)	0.0135 (16)
C21	0.105 (2)	0.0768 (14)	0.111 (2)	−0.0185 (14)	0.0625 (17)	0.0020 (14)
C22	0.111 (2)	0.0696 (13)	0.0735 (14)	−0.0183 (13)	0.0308 (13)	0.0041 (11)
C23	0.0760 (13)	0.0611 (11)	0.0788 (13)	−0.0049 (10)	0.0220 (11)	0.0003 (10)
S1B	0.0739 (16)	0.0672 (17)	0.057 (2)	0.0128 (13)	0.0078 (14)	−0.0085 (15)
C13B	0.061 (6)	0.167 (16)	0.062 (8)	−0.032 (7)	−0.009 (5)	0.011 (8)

### Geometric parameters (Å, °)

**Table:**

S1A—C12	1.693 (2)	C18—C23	1.384 (3)
S1A—C15	1.649 (3)	C18—C19	1.381 (4)
S1B—C14	1.580 (6)	C19—C20	1.389 (4)
S1B—C12	1.664 (6)	C20—C21	1.347 (4)
O1—C7	1.2339 (19)	C21—C22	1.359 (4)
O2—C10	1.171 (3)	C22—C23	1.383 (4)
O3—C11	1.455 (3)	C2—H2	0.9300
O3—C10	1.309 (3)	C3—H3	0.9300
N1—C7	1.347 (2)	C4—H4	0.9300
N1—C8	1.465 (2)	C5—H5	0.9300
N1—C16	1.466 (2)	C8—H8	0.9800
C1—C2	1.390 (3)	C9—H9	0.9800
C1—C9	1.508 (3)	C11—H11A	0.9600
C1—C6	1.395 (2)	C11—H11B	0.9600
C2—C3	1.385 (3)	C11—H11C	0.9600
C3—C4	1.387 (3)	C13A—H13A	0.9300
C4—C5	1.373 (3)	C13B—H13B	0.9300
C5—C6	1.391 (3)	C14—H14	0.9300
C6—C7	1.492 (2)	C15—H15	0.9300
C8—C12	1.514 (3)	C16—H16A	0.9700
C8—C9	1.529 (3)	C16—H16B	0.9700
C9—C10	1.543 (3)	C17—H17B	0.9700
C12—C13B	1.379 (15)	C17—H17A	0.9700
C12—C13A	1.363 (8)	C19—H19	0.9300
C13A—C14	1.415 (10)	C20—H20	0.9300
C13B—C15	1.478 (15)	C21—H21	0.9300
C14—C15	1.328 (3)	C22—H22	0.9300
C16—C17	1.510 (3)	C23—H23	0.9300
C17—C18	1.505 (4)		
			
S1A···N1	2.980 (2)	C14···H3^iii^	2.9700
S1A···C6	3.542 (2)	C15···H3^iii^	3.0400
S1A···C7	3.325 (2)	C16···H19	3.0000
S1A···C16	3.584 (3)	C17···H8	2.9100
S1A···S1A^i^	3.636 (2)	C19···H16B	2.8800
S1B···H16A^ii^	3.0400	C20···H11B^x^	3.0400
S1B···H3^iii^	3.1200	C21···H11B^xiii^	3.0700
S1B···H9	3.0400	C21···H11C^x^	3.0300
O1···C15^i^	3.420 (3)	C22···H8^iv^	2.8000
O1···C2^iv^	3.401 (3)	C23···H22^xii^	3.0800
O2···N1	3.023 (2)	H2···O1^ii^	2.5700
O2···C17	3.269 (3)	H2···H9	2.4800
O3···C2	3.214 (3)	H3···C12^v^	3.0900
O1···H20^v^	2.6600	H3···C15^v^	3.0400
O1···H16A	2.3200	H3···S1B^v^	3.1200
O1···H15^i^	2.5500	H3···C13B^v^	3.0200
O1···H5	2.5500	H3···C14^v^	2.9700
O1···H2^iv^	2.5700	H3···C13A^v^	2.9500
O2···H11C	2.5400	H5···O1	2.5500
O2···H11A^vi^	2.8300	H8···O2	2.5400
O2···H17A	2.8600	H8···H16B	2.5200
O2···H11A	2.6200	H8···H17B	2.4700
O2···H8	2.5400	H8···C17	2.9100
O2···H22^ii^	2.8800	H8···C22^ii^	2.8000
O3···H21^vii^	2.7800	H9···C13A	3.0600
N1···O2	3.023 (2)	H9···S1B	3.0400
N1···S1A	2.980 (2)	H9···H2	2.4800
N1···H13B	2.8000	H11A···O2	2.6200
C2···O1^ii^	3.401 (3)	H11A···O2^xii^	2.8300
C2···O3	3.214 (3)	H11B···H21^vii^	2.3200
C4···C4^viii^	3.540 (3)	H11B···C20^ix^	3.0400
C5···C19^v^	3.553 (3)	H11B···C21^vii^	3.0700
C6···C12	3.450 (2)	H11B···H11C^xii^	2.4300
C6···S1A	3.542 (2)	H11C···C11^vi^	2.9500
C7···S1A	3.325 (2)	H11C···O2	2.5400
C7···C13B	3.472 (16)	H11C···H11B^vi^	2.4300
C7···C10	3.433 (3)	H11C···C21^ix^	3.0300
C10···C7	3.433 (3)	H13B···N1	2.8000
C10···C23^ii^	3.550 (3)	H13B···C7	2.9900
C11···C21^ix^	3.487 (4)	H14···C3^xi^	3.0900
C11···C20^ix^	3.446 (4)	H15···O1^i^	2.5500
C12···C6	3.450 (2)	H15···C2^xi^	3.0900
C13B···C7	3.472 (16)	H16A···S1B^iv^	3.0400
C15···O1^i^	3.420 (3)	H16A···O1	2.3200
C16···S1A	3.584 (3)	H16B···C3^iii^	3.1000
C17···O2	3.269 (3)	H16B···C4^iii^	3.0800
C19···C5^iii^	3.553 (3)	H16B···C12	2.9200
C20···C11^x^	3.446 (4)	H16B···C19	2.8800
C21···C11^x^	3.487 (4)	H16B···H8	2.5200
C23···C10^iv^	3.550 (3)	H16B···H19	2.5300
C2···H15^xi^	3.0900	H17A···H23	2.3300
C3···H16B^v^	3.1000	H17A···O2	2.8600
C3···H14^xi^	3.0900	H17B···C8	3.0900
C4···H16B^v^	3.0800	H17B···H8	2.4700
C4···H19^v^	3.0900	H19···H16B	2.5300
C5···H19^v^	3.0200	H19···C4^iii^	3.0900
C6···H20^v^	3.0800	H19···C5^iii^	3.0200
C7···H20^v^	2.9500	H19···C16	3.0000
C7···H13B	2.9900	H20···C6^iii^	3.0800
C8···H17B	3.0900	H20···C7^iii^	2.9500
C10···H23^ii^	3.0400	H20···O1^iii^	2.6600
C11···H21^vii^	2.9600	H21···O3^xiii^	2.7800
C11···H11C^xii^	2.9500	H21···C11^xiii^	2.9600
C12···H3^iii^	3.0900	H21···H11B^xiii^	2.3200
C12···H16B	2.9200	H22···C23^vi^	3.0800
C13A···H9	3.0600	H22···O2^iv^	2.8800
C13A···H3^iii^	2.9500	H23···C10^iv^	3.0400
C13B···H3^iii^	3.0200	H23···H17A	2.3300
			
C12—S1A—C15	92.90 (12)	C1—C2—H2	120.00
C12—S1B—C14	93.3 (3)	C3—C2—H2	120.00
C10—O3—C11	116.0 (2)	C2—C3—H3	120.00
C7—N1—C16	119.22 (15)	C4—C3—H3	120.00
C8—N1—C16	117.38 (15)	C3—C4—H4	120.00
C7—N1—C8	122.88 (15)	C5—C4—H4	120.00
C2—C1—C6	119.50 (17)	C4—C5—H5	120.00
C2—C1—C9	122.53 (15)	C6—C5—H5	120.00
C6—C1—C9	117.94 (15)	N1—C8—H8	107.00
C1—C2—C3	119.89 (19)	C9—C8—H8	107.00
C2—C3—C4	120.27 (18)	C12—C8—H8	107.00
C3—C4—C5	120.22 (19)	C1—C9—H9	109.00
C4—C5—C6	120.07 (19)	C8—C9—H9	109.00
C1—C6—C5	120.05 (17)	C10—C9—H9	109.00
C1—C6—C7	120.76 (16)	O3—C11—H11A	109.00
C5—C6—C7	119.19 (15)	O3—C11—H11B	110.00
O1—C7—N1	122.15 (17)	O3—C11—H11C	109.00
O1—C7—C6	121.05 (16)	H11A—C11—H11B	109.00
N1—C7—C6	116.80 (13)	H11A—C11—H11C	109.00
N1—C8—C12	111.38 (14)	H11B—C11—H11C	109.00
C9—C8—C12	113.24 (14)	C12—C13A—H13A	122.00
N1—C8—C9	110.23 (14)	C14—C13A—H13A	122.00
C1—C9—C8	110.38 (14)	C12—C13B—H13B	122.00
C1—C9—C10	110.76 (15)	C15—C13B—H13B	122.00
C8—C9—C10	110.01 (15)	C13A—C14—H14	126.00
O2—C10—O3	123.0 (2)	C15—C14—H14	126.00
O2—C10—C9	125.87 (19)	S1B—C14—H14	113.00
O3—C10—C9	111.10 (17)	S1A—C15—H15	122.00
S1A—C12—C13A	107.5 (4)	C13B—C15—H15	136.00
C8—C12—C13A	130.6 (4)	C14—C15—H15	122.00
S1A—C12—C8	121.91 (12)	N1—C16—H16B	109.00
C8—C12—C13B	131.4 (6)	N1—C16—H16A	109.00
S1B—C12—C13B	107.4 (7)	H16A—C16—H16B	108.00
S1B—C12—C8	121.1 (2)	C17—C16—H16A	109.00
C12—C13A—C14	116.4 (6)	C17—C16—H16B	109.00
C12—C13B—C15	115.9 (10)	C18—C17—H17A	110.00
C13A—C14—C15	107.6 (4)	C18—C17—H17B	110.00
S1B—C14—C15	121.2 (3)	H17A—C17—H17B	108.00
C13B—C15—C14	102.1 (6)	C16—C17—H17A	109.00
S1A—C15—C14	115.60 (18)	C16—C17—H17B	109.00
N1—C16—C17	114.22 (18)	C18—C19—H19	120.00
C16—C17—C18	110.76 (19)	C20—C19—H19	120.00
C17—C18—C19	122.0 (2)	C19—C20—H20	120.00
C19—C18—C23	117.5 (2)	C21—C20—H20	120.00
C17—C18—C23	120.4 (2)	C22—C21—H21	120.00
C18—C19—C20	120.8 (3)	C20—C21—H21	120.00
C19—C20—C21	120.1 (3)	C21—C22—H22	120.00
C20—C21—C22	120.7 (3)	C23—C22—H22	120.00
C21—C22—C23	119.7 (2)	C22—C23—H23	119.00
C18—C23—C22	121.1 (2)	C18—C23—H23	119.00
			
C15—S1A—C12—C8	178.77 (17)	C5—C6—C7—O1	15.7 (3)
C12—S1A—C15—C14	0.3 (2)	C5—C6—C7—N1	−164.20 (18)
C15—S1A—C12—C13A	0.0 (5)	C1—C6—C7—N1	16.6 (3)
C11—O3—C10—C9	−176.0 (2)	N1—C8—C9—C10	−70.98 (18)
C11—O3—C10—O2	5.8 (4)	C12—C8—C9—C1	−73.97 (17)
C16—N1—C7—C6	175.20 (16)	C12—C8—C9—C10	163.51 (14)
C8—N1—C7—C6	3.6 (3)	N1—C8—C12—S1A	−19.2 (2)
C8—N1—C16—C17	−81.0 (2)	N1—C8—C9—C1	51.54 (19)
C16—N1—C7—O1	−4.7 (3)	C9—C8—C12—C13A	−75.9 (6)
C7—N1—C16—C17	107.0 (2)	N1—C8—C12—C13A	159.2 (6)
C7—N1—C8—C9	−38.0 (2)	C9—C8—C12—S1A	105.70 (17)
C8—N1—C7—O1	−176.20 (17)	C8—C9—C10—O2	1.7 (3)
C16—N1—C8—C12	−83.1 (2)	C1—C9—C10—O2	−120.6 (2)
C16—N1—C8—C9	150.35 (16)	C8—C9—C10—O3	−176.42 (17)
C7—N1—C8—C12	88.6 (2)	C1—C9—C10—O3	61.3 (2)
C6—C1—C2—C3	0.4 (3)	S1A—C12—C13A—C14	−0.4 (8)
C9—C1—C6—C7	1.1 (3)	C8—C12—C13A—C14	−179.0 (4)
C2—C1—C9—C8	146.92 (17)	C12—C13A—C14—C15	0.6 (9)
C2—C1—C9—C10	−91.0 (2)	C13A—C14—C15—S1A	−0.6 (5)
C9—C1—C6—C5	−178.09 (17)	N1—C16—C17—C18	−168.81 (19)
C9—C1—C2—C3	178.27 (18)	C16—C17—C18—C19	−61.7 (3)
C2—C1—C6—C5	−0.1 (3)	C16—C17—C18—C23	113.8 (2)
C2—C1—C6—C7	179.10 (17)	C17—C18—C19—C20	172.5 (2)
C6—C1—C9—C10	86.9 (2)	C23—C18—C19—C20	−3.2 (3)
C6—C1—C9—C8	−35.1 (2)	C17—C18—C23—C22	−174.2 (2)
C1—C2—C3—C4	−0.4 (3)	C19—C18—C23—C22	1.5 (3)
C2—C3—C4—C5	0.1 (3)	C18—C19—C20—C21	2.2 (4)
C3—C4—C5—C6	0.2 (3)	C19—C20—C21—C22	0.5 (4)
C4—C5—C6—C1	−0.2 (3)	C20—C21—C22—C23	−2.2 (4)
C4—C5—C6—C7	−179.38 (18)	C21—C22—C23—C18	1.1 (3)
C1—C6—C7—O1	−163.54 (18)		

Symmetry codes: (i) −*x*+2, −*y*, −*z*+1; (ii) *x*, *y*, *z*+1; (iii) *x*+1, *y*, *z*; (iv) *x*, *y*, *z*−1; (v) *x*−1, *y*, *z*; (vi) *x*, −*y*+1/2, *z*−1/2; (vii) *x*−1, *y*, *z*+1; (viii) −*x*+1, −*y*, −*z*+1; (ix) *x*−1, −*y*+1/2, *z*+1/2; (x) *x*+1, −*y*+1/2, *z*−1/2; (xi) −*x*+2, −*y*, −*z*+2; (xii) *x*, −*y*+1/2, *z*+1/2; (xiii) *x*+1, *y*, *z*−1.

### Hydrogen-bond geometry (Å, °)

**Table:**

*D*—H···*A*	*D*—H	H···*A*	*D*···*A*	*D*—H···*A*
C2—H2···O1^ii^	0.93	2.57	3.401 (3)	149
C15—H15···O1^i^	0.93	2.55	3.420 (3)	155
C16—H16A···O1	0.97	2.32	2.731 (2)	105
C3—H3···Cg1^v^	0.93	2.78	3.688 (3)	165
C3—H3···Cg2^v^	0.93	2.77	3.688 (4)	167
C19—H19···Cg3^iii^	0.93	2.89	3.692 (3)	145

Symmetry codes: (ii) *x*, *y*, *z*+1; (i) −*x*+2, −*y*, −*z*+1; (v) *x*−1, *y*, *z*; (iii) *x*+1, *y*, *z*.
